# miR-103a-3p Silencing Ameliorates Calcium Oxalate Deposition in Rat Kidney by Activating the UMOD/TRPV5 Axis

**DOI:** 10.1155/2022/2602717

**Published:** 2022-02-23

**Authors:** Zenglin Cui, Yuwei Li, Gaorui Liu, Yanmeng Jiang

**Affiliations:** ^1^Department of Urology, The Third Affiliated Hospital of Xinxiang Medical University, Xinxiang, China; ^2^Endoscopy Department, The Third Affiliated Hospital of Xinxiang Medical University, Xinxiang, China

## Abstract

Maintaining the balance of calcium (Ca^2+^) metabolism in the kidney is crucial in preventing the formation of kidney stones. Functionally, the microRNA (miRNA) participating in this process needs to be unveiled. We induced NRK-52E cell injury by oxalate treatment. The role of transient receptor potential cation channel subfamily V member 5 (TRPV5) in oxalate-induced cells was studied by TRPV5 overexpression transfection, qRT-PCR, Western blot, MTT, and crystal adhesion detection. After identifying uromodulin (UMOD) expression in injured cells, we confirmed the interaction between TRPV5 and UMOD by coimmunoprecipitation (CoIP) and cell-surface biotinylation assays. The validation of UMOD-regulating TRPV5 in viability, crystal adhesion, and Ca^2+^ concentration of oxalate-induced cells was performed. Bioinformatics analysis and luciferase assay were used to identify the miRNA-targeting UMOD. The role of the miR-103a-3p-regulating UMOD/TRPV5 axis was detected by rescue experiments. We constructed a rat model with treatment of ethylene glycol (EG) to investigate the miR-103a-3p/UMOD/TRPV5 axis *in vivo* by hematoxylin-eosin (H&E) staining, Western blot, and immunohistochemistry (IHC). Upregulation of TRPV5 protected NRK-52E cells from oxalate-induced injury by enhancing cell viability and inhibiting CaOx adhesion. UMOD was depleted in oxalate-induced cells and positively interacted with TRPV5. UMOD silencing reversed the effect of TRPV overexpression on oxalate-induced cells. miR-103a-3p targeted UMOD and was mediated in the regulation of the UMOD/TRPV5 axis in oxalate-induced cells. Downregulating miR-103a-3p mitigated EG-induced CaOx deposition in kidney tissues *in vivo* by activating the UMOD/TRPV5 axis. miR-103a-3p silencing ameliorated CaOx deposition in the rat kidney by activating the UMOD/TRPV5 axis.

## 1. Introduction

Urinary calculi, one of the most common diseases in urology, are caused by the precipitation of oversaturated crystals from the urine in the kidney [1]. Although not as aggressive as malignant tumors, urinary calculi have long had a serious impact on human health due to their high incidence and recurrence rate [2]. Studies have confirmed that the onset of urinary calculi is multifactorial and involves genetics, metabolism, environmental climate, and lifestyle habits [3, 4]. Calcium oxalate (CaOx) stones account for the majority of cases of this disease, and most patients with Ca^2+^-containing stones have a combination of hypercalciuria [5]. Physicochemical studies have verified that the saturation of CaOx in urine is related to the concentration of Ca^2+^ and that increased urinary calcium predisposes to or promotes the formation of Ca^2+^-containing nephrolithiasis [6]. Kidney stones can lead to hypertension, chronic kidney disease, and end-stage renal disease. Studies have reported that preventive measures, including specific pharmacological interventions and recommendations for lifestyle and nutritional changes, can be pursued after a comprehensive metabolic assessment [7, 8]. Various human studies have suggested that diets with a higher intake of vegetables and fruits play a role in the prevention of kidney stones [9–11]. At present, an in-depth study should be conducted on the molecular mechanism of hypercalciuria induced by abnormal calcium metabolism in the formation of kidney stones.

TRPV5 is an important protein that regulates the transport of Ca^2+^ across cell membranes [12]. In renal tissues, TRPV5 is mainly found in the distal convoluted tubule (DCT) and collecting tubule (CNT) that is responsible for regulating urinary Ca^2+^ reabsorption and maintaining Ca^2+^ homeostasis in the body [13]. Available evidence suggests that downregulated TRPV5 expression may be a vital pathogenic factor in the formation of hypercalciuria and Ca^2+^-containing nephrolithiasis [14].

Uromodulin (UMOD), also known as Tamm-Horsfall protein, is the most abundant glycoprotein in urine [15], which is primarily expressed in renal tubular epithelial cells with a variety of physiological functions such as balancing the water-electrolyte metabolism, regulating immunity, preventing kidney stones, and protecting against urinary tract infections [16–18]. At present, there are few reports on the specific molecular mechanism of UMOD in kidney stone formation.

MicroRNAs (miRNAs) are a class of small, noncoding, single-stranded RNA molecules with a length of about 20–22 nucleotides, widely existing in mammals [19]. Mature miRNAs can regulate the post-transcriptional expression of target genes by binding fully or partially complementarily to the 3′-UTR of the target mRNA, resulting in the degradation or inhibition of protein translational synthesis [20]. Since miRNAs dramatically affect human physiological activities such as growth and development, and cell apoptosis and metabolism, they are considered to hold great promise for the prevention and treatment of diseases [21]. In recent years, research concerning the role of miRNAs in the onset and development of kidney stones has become a hot topic [22, 23].

Therefore, this study probed into the relationship between UMOD and TRPV5 in the prevention of kidney stone formation and elucidated the role of miR-103a-3p in targeting and regulating UMOD, contributing to a deeper understanding of the pathogenesis of kidney stones and providing new ideas for the prevention and management of the disease.

## 2. Material and Methods

### 2.1. Cell Culture and Oxalate Treatment

Epithelioid rat kidney cell line NRK-52E (GNR 8) was obtained from the China Center for Type Culture Collection (CCTCC) (Shanghai, China). DMEM (12800017, Gibco, USA) mixed with distilled water, 7.5% NaHCO_3_ solution (25080094, Gibco, USA), and 5% fetal bovine serum (FBS) (BC-SE-FBS02, Biochannel, China) was prepared to culture cells in an incubator (37°C and 5% CO_2_). After subculturing, the cells were induced with diluted oxalate (0.75 mmol/L, 135623, Sigma-Aldrich, USA) for 48 hours (h), as described previously [24].

### 2.2. Cell Transfection

For the molecular mechanism study, TRPV5-overexpressing plasmid (Bes-mR-001007572, BersinBio, Guangzhou, China), UMOD-overexpressing plasmid (Bes-mR-001278605, BersinBio, Guangzhou, China), small interfering RNA against UMOD (siUMOD) (5′-CTCTTTTATCACACTTGATATGA-3′, GenePharma, Shanghai, China), miR-103a-3p inhibitor (GenePharma, Shanghai, China), and miR-103a-3p mimic (BersinBio, Guangzhou, China) were subjected to different transfections in NRK-52E cells, with corresponding controls being set up. Before transfection, NRK-52E cells were cultured in a 6-well plate (2 × 10^6^ cells/well). Until reaching about 80% cell confluence, TRPV5-overexpressing plasmid, UMOD-overexpressing plasmid, siUMOD, miR-103a-3p inhibitor, or mimic was transfected into NRK-52E cells using lipofectamine 2000 (11668019, Invitrogen, USA) according to the manufacturer's protocol. After that, the transfected NRK-52E cells received quantitative real-time polymerase chain reaction (qRT-PCR) and Western blot for quality assessment and then were treated with oxalate.

### 2.3. RNA Extraction and qRT-PCR

Total RNA and miRNA from NRK-52E cells were extracted using Cell RNA Kit (19231ES50, Yeasen, China) and MolPure® Cell/Tissue miRNA Kit (19331ES50, Yeasen, China). Then, HiScript II One Step RT-PCR Kit (P612-01, Vazyme, China) was employed to react with the extract following the operating instructions. The amplification condition was set as follows: 50°C for 30 min, 94°C for 3 min, followed by 30 cycles (94°C for 30 s, 60°C for 30 s, and 72°C for 40 s), and 72°C for 5 min. The qRT-PCR was conducted in the ABI 7500 system (Applied Biosystems, USA). Relative mRNA expressions were analyzed by the 2^−ΔΔ*Ct*^ method [25], with *β*-actin or U6 using for normalization. All primers sequences were shown in Table 1.

### 2.4. Western Blot

Total protein from samples (NRK-52E cells and rat kidney tissues) was lysed using the RIPA Lysis Buffer (R0010, Solarbio, China) and quantified by the BCA Protein Assay Kit (BI-WB005, Sbjbio, China). Equal amounts of protein were separated by 10% sodium dodecyl sulfate polyacrylamide gel electrophoresis (P0690, Beyotime, China) and transferred onto PVDF membranes (AR0136-02, Boster, China). After being treated with blocking buffer (P0252, Beyotime, China), the membranes were incubated with anti-TRPV5 (ab137028, 83 kDa, 1/10000), anti-UMOD (ab207170, 110 kDa, 1/1000), and anti-*β*-actin (ab8226, 42 kDa, 1/1000) antibodies at 4°C overnight. The secondary antibodies including anti-rabbit IgG (ab97051, 1/2000) and anti-mouse IgG (ab205719, 1/2000) were then used to incubate the membranes for 2 h at room temperature. Relative protein blot detection was run the in iBright Imaging System (CL750, Invitrogen, USA) by using ECL Buffer (WBKlS0100, Millipore, USA) for visualization. *β*-Actin served as the internal control.

### 2.5. Cell Viability Assay

Cell suspensions were prepared as described in this study [24]. NRK-52E cells were seeded in 96-well culture plates at a density of 5000 cells/well, and 10 *μ*L methylthiazolyldiphenyl-tetrazolium bromide (MTT) solution (40201ES72, Yeasen, China) was used to treat the cells in each well at 37°C for 4 h, followed by SDS-HCL treatment. For cell viability evaluation, the optical density (OD) value at a wavelength of 570 nm was measured by a microplate reader (VL0L00D0, Thermo Fisher, USA).

### 2.6. Crystal Cell Adhesion Detection

After oxalate treatment, NRK-52E cells (2 × 10^5^ cells/well) were resuspended in a 6-well plate for obtaining over 90% cell confluence. Then, the cells were washed with PBS and intubated in DMEM containing 40 *μ*g/mL with Ponceau S-labelled CaOx monohydrate (COM) crystals at room temperature for 10 minutes (min). After being washed multiple times with PBS to remove unbound COM crystals, images were captured by an inverted microscope (IX-71, Olympus, Japan) and the number of adherent crystals in 10 randomized fields was calculated.

### 2.7. Coimmunoprecipitation (CoIP)

The physiological interaction between TRPV5 and UMOD was detected using CoIP Kit (abs955, Absin, China) in NRK-52E cells transfected with TRPV5-Flag and UMOD. Briefly, cell lysates (500 *μ*L) were prepared as per the manufacturer's instructions and incubated with antibodies of TRPV5 (1 *μ*L) and UMOD (1 *μ*L) at 4°C overnight. Then, 5 *μ*L protein A and G beads washed with lysis buffer were used to treat the lysates at 4°C for 3 h. Afterwards, the immunoprecipitates were washed 3 times with wash buffer and resuspended with 1 × SDS loading buffer (20 *μ*L). After 5 min of heating, the immunoprecipitates were subjected to Western blot.

### 2.8. Cell-Surface Biotinylation Assay

The enrichment of TRPV5 on the surface of NRK-52E cells was examined using the Cell Surface Biotinylation Kit (A44390, Pierce, USA). The transfected cells in suspension (6 × 10^5^ cells/mL) were washed with PBS and incubated with EZ-Link-NHS-SS-biotin solution at room temperature for 10 min. Next, the cells were lysed with lysis buffer, with labeled proteins isolated with NeutrAvidin Agarose as the protocols guided. Elution buffer mixed with Dithithreitol stock solution was used to incubate isolated proteins at room temperature for 30 min, followed by 2 min of centrifugation. Finally, the eluate was measured by Western blot. Total lysate TRPV5 was determined simultaneously as a negative control, and *β*-actin served as the internal control.

### 2.9. Ca^2+^ Content Measurement

The Fluo-4 AM Kit (S1060, Beyotime, China) was applied to evaluate intracellular Ca^2+^ concentration. This experimental procedure refers to the previous study [26]. NRK-52E cells were subjected to multiple rinses and then probed with PBS-diluted Fluo-4 AM at 37°C. After 30 min, a laser scanning confocal microscopy (FV500-IX71, Olympus, Japan) was used to monitor Fluo-4 fluorescence excited at 494 nm. The value of fluorescent intensity was recorded to calculate the concentration ([Ca^2+^]_i_).

### 2.10. Bioinformatics Analysis and Luciferase Assay

UMOD-targeting miRNA, miR-103a-3p, was predictably analyzed by TargetScan website (http://www.targetscan.org/vert_71/) and then was validated via luciferase assay. pGL3-control vectors (E1741) were purchased from Promega (USA) to construct the wild type of UMOD (UMOD-WT, 5′-AGCCUGUGUCUUUAAAUGCUGCU-3′) and the mutant type of UMOD (UMOD-MUT, 5′-AGCCUGUGUCUUUAAAUGAUCCU-3′). NRK-52E cells were cotransfected with miR-103a-3p mimic and UMOD-WT or miR-103a-3p mimic and UMOD-MUT with the help of Lipofectamine 2000. The identical procedure was performed in the mimic control (pRL-TK vectors, VT1568, YouBio, China). After that, we determined luciferase activity in cells using the Dual-Luciferase Reporter Assay Kit (E1910, Promega, USA).

### 2.11. Hyperoxaluria Model Establishment *In Vivo*

For the *in vivo* study, we established the hyperoxaluria rat model by adding 1% ethylene glycol (EG) (324558, Sigma-Aldrich, USA) in drinking water. The animal experiment was approved by the Committee of Laboratory Animals of The Third Affiliated Hospital of Xinxiang Medical University (IHRM20200510). A total of 40 male Sprague Dawley (SD) rats (8 weeks old, 180–210 g) purchased from Charles River Laboratories (Beijing, China) were used in *in vivo* study. All rats were divided into 4 groups of control, EG, EG + antagomir, and EG + antagomir-NC, with ten rats in each group. During housing, in the control group, the rats were only provided with tap water; in the EG group, the rats were treated with 1% EG; in the EG + antagomir group, the rats were treated with 1% EG and intravenously injected with miR-103a-3p antagomir; and in the EG + antagomir-NC group, the rats were treated with 1% EG and intravenously injected with miR-103a-3p antagomir-NC. After 4 weeks, all rats were sacrificed by cervical dislocation under anesthesia (pentobarbital sodium, 40 mg/kg, P-010, Merck, USA) and kidney tissues were collected for histological study. The miR-103a-3p antagomirs used in this experiment were obtained from Ribobio (Guangzhou, China).

### 2.12. Hematoxylin-Eosin (HE) Staining

Formalin-fixed kidney tissues were embedded in paraffin for CaOx deposition assessment. Sectioned samples were dewaxed and hydrated prior to the HE staining. Then, the HE Staining Kit (C0105S, Beyotime, China) was used to treat the samples guided by the operating instructions. After sealing the tissues, the crystal deposition area was observed under a polarizing microscope (Axio Scope.A1, Precise Instrument, China) at ×100 magnification and was quantified using ImageJ (vision 1.8.0, National Institutes of Health, USA).

### 2.13. Immunohistochemistry (IHC)

In order to study the distribution of TRPV5 in renal tissues, we used the Super Plus™ High Sensitive and Rapid Immunohistochemical Kit (E-IR-R221, Elabscience, China) to conduct IHC. In short, kidney sections (4 *μ*m) were treated with Dewaxing/Antigen Retrieval Buffer for 30 min in heating and subsequently incubated with SP Reagent B Peroxidase Blocking Buffer at room temperature for 15 min. After PBS washing, the sections were reacted with anti-TRPV5 antibody (ab137028, 1/100, Abcam, UK) at 37°C for 2 h, followed by secondary antibody incubation. Thereafter, the sections were required for color development in DAB working solution and staining in hematoxylin buffer. After rebluing treatment, the staining images were captured using a microscope (CX23, Olympus, Japan) at ×100 magnification.

### 2.14. Statistical Analysis

All experiments were repeated three times individually, with measurement data describing as the mean ± standard deviation. Differences compared between multiple groups were analyzed by one-way analysis of variance. GraphPad Prism 8.0 (GraphPad Software, USA) was used for data analysis, and a *p* value < 0.05 indicated a statistically significant difference.

## 3. Results

### 3.1. Upregulation of TRPV5 Protected NRK-52E Cells from Oxalate-Induced Injury

**Figure 1:**
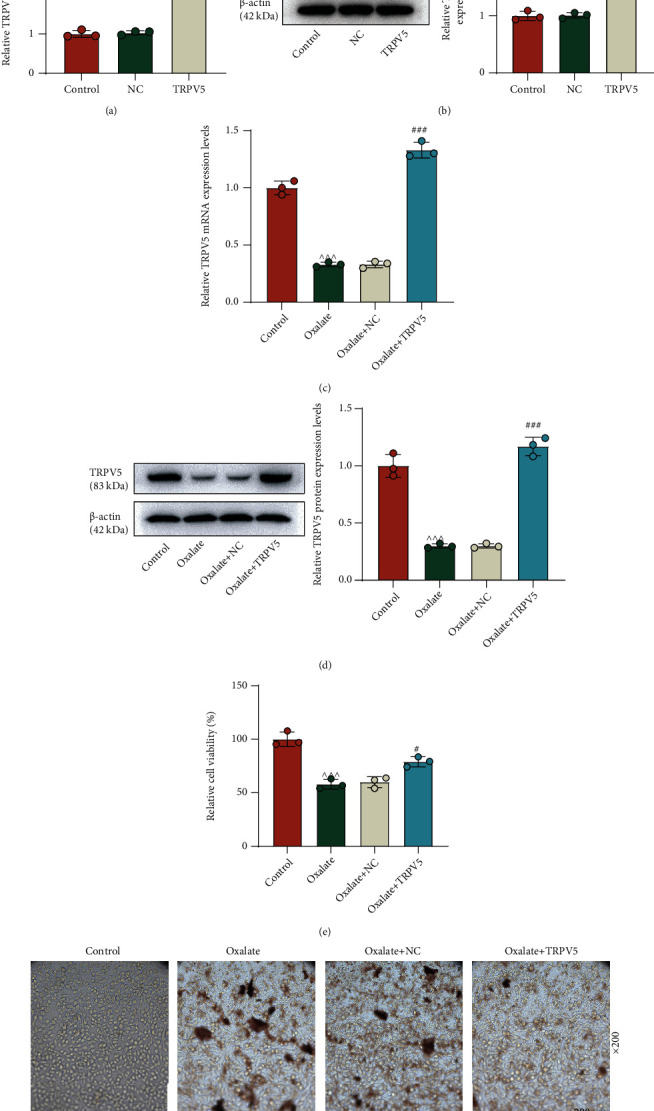
The effect of TRPV5 on oxalate-induced NRK-52E cells. (a) QRT-PCR was performed to detect the mRNA expression of TRPV5 in NRK-52E cells after TRPV5 overexpression. *β*-Actin served as the internal control. (b) Western blot was performed to measure the protein level of TRPV5 in NRK-52E cells after TRPV5 overexpression. *β*-Actin served as the internal control. (c) The expression of TRPV5 in oxalate-induced NRK-52E cells with or without TRPV5 overexpression was detected by qRT-PCR. *β*-Actin served as the internal control. (d) The protein level of TRPV5 in oxalate-induced NRK-52E cells with or without TRPV5 overexpression was measured by Western blot. *β*-Actin served as the internal control. (e) MTT assay was conducted to evaluate cell viability. (f) Crystal cell adhesion assay was used to determine and quantify CaOx deposition in specified cells (×200 magnification, scale bar = 200 *μ*m). ^∗∗∗^*p* < 0.001 vs. NC; ^∧∧∧^*p* < 0.001 vs. control; ^#^*p* < 0.05 and ^###^*p* < 0.001 vs. oxalate + NC. TRPV5: transient receptor potential cation channel subfamily V member 5; qRT-PCR: quantitative real-time polymerase chain reaction; MTT: methylthiazolyldiphenyl-tetrazolium bromide; NC: negative control.

Based on the pivotal role of TRPV5 in regulating Ca^2+^ transport in renal tubular cells, we first performed TRPV5 overexpression in NRK-52E cells. As demonstrated in Figure 1(a), a significant increase of the TRPV5 mRNA level was detected by qRT-PCR (*p* < 0.001). Western blot has also measured the high level of TRPV5 protein in transfected cells (Figure 1(b), *p* < 0.001). After oxalate treatment in NRK-52E cells, we found that the expression of TRPV5 was downregulated compared to that of the control group, which was reversed by transfecting TRPV5-overexpressing plasmid (Figure 1(c), *p* < 0.001). In addition, the detection of Western blot presented similar outcomes (Figure 1(d), *p* < 0.001). The following MTT assay indicated that oxalate attenuated cell viability, which was partially overturned after TRPV5 overexpression (Figure 1(e), *p* < 0.05). The detection of crystal adhesion showed that TRPV5 overexpression strikingly inhibited the adhesion of CaOx induced by oxalate in NRK-52E cells (Figure 1(f)).

### 3.2. Oxalate Induction Downregulated UMOD Expression which Was Positively Associated with the Enrichment of TRPV5 on the Cell Surface

**Figure 2:**
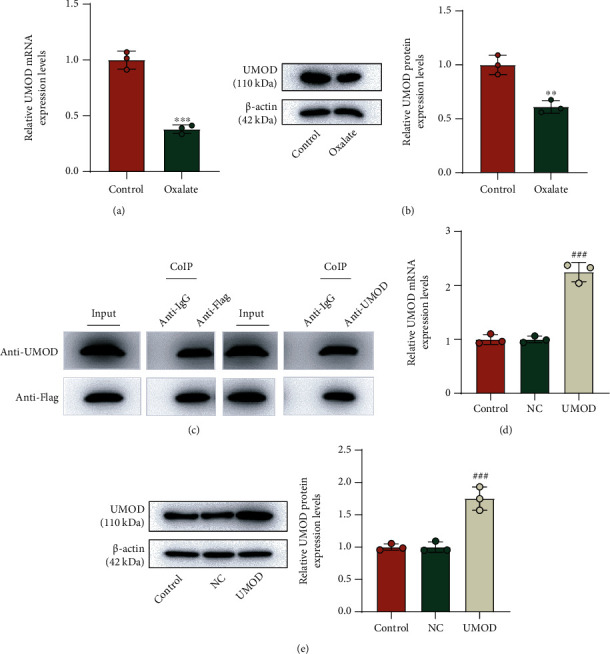
The expression of UMOD in oxalate-induced NRK-52E cells and its physiological interaction with TRPV5. (a) QRT-PCR was performed to detect the mRNA expression of UMOD in oxalate-induced NRK-52E cells. *β*-Actin served as the internal control. (b) Western blot was performed to measure the protein level of UMOD in oxalate-induced NRK-52E cells. *β*-Actin served as the internal control. (c) CoIP was used to detect the physiological interaction between TRPV5 and UMOD. (d) After UMOD overexpression in NRK-52E cells, qRT-PCR was used to verify the expression of UMOD. *β*-Actin served as the internal control. (e) The UMOD protein level was verified using Western blot. *β*-Actin served as the internal control. ^∗∗^*p* < 0.01 and ^∗∗∗^*p* < 0.001 vs. control; ^###^*p* < 0.001 vs. NC. UMOD: uromodulin; qRT-PCR: quantitative real-time polymerase chain reaction; CoIP: coimmunoprecipitation; NC: negative control.

**Figure 3:**
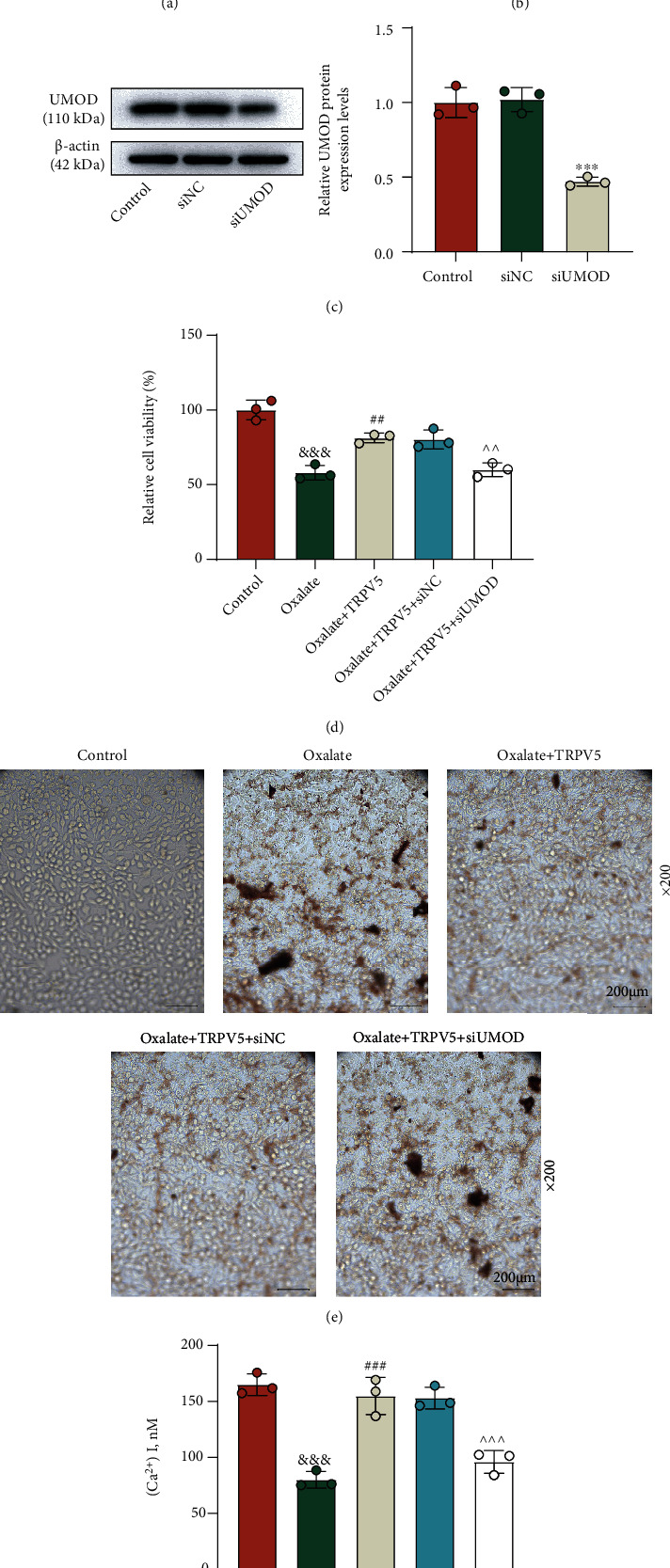
The regulating role of the UMOD/TRPV5 axis in oxalate-induced NRK-52E cells. (a) Cell-surface biotinylation assay was applied to assess the enrichment of TRPV5 on the surface of NRK-52E cells after UMOD overexpression. (b) QRT-PCR was used to verify the mRNA expression of UMOD after siUMOD transfection. *β*-Actin served as the internal control. (c) Western blot was performed to verify the protein level of UMOD after siUMOD transfection. *β*-Actin served as the internal control. (d) MTT was used to measure cell viability following the treatment with oxalate, TRPV5-overexpressing plasmid, and siUMOD in NRK-52E cells. (e) Crystal cell adhesion assay was used to determine and quantify CaOx deposition in specified cells (×200 magnification, scale bar = 200 *μ*m). (f) The Ca^2+^ content measurement in different groups was analyzed by Fluo-4 AM Kit. ^∗∗∗^*p* < 0.001 vs. siNC; ^&&&^*p* < 0.001 vs. control; ^##^*p* < 0.01 and ^###^*p* < 0.001 vs. oxalate; ^∧∧^*p* < 0.01 and ^∧∧∧^*p* < 0.001 vs. oxalate + TRPV5 + siNC. TRPV5: transient receptor potential cation channel subfamily V member 5; UMOD: uromodulin; qRT-PCR: quantitative real-time polymerase chain reaction; MTT: methylthiazolyldiphenyl-tetrazolium bromide; NC: negative control; siUMOD: small interfering RNA against UMOD.

The mRNA expression of UMOD was found to be downregulated in oxalate-induced NRK-52E cells (Figure 2(a), *p* < 0.001), so as its protein level (Figure 2(b), *p* < 0.01). The results of CoIP verified that UMOD was considerably coimmunoprecipitated by anti-flag-tagged TRPV5, and conversely, TRPV5 was coimmunoprecipitated by anti-UMOD (Figure 2(c)). Next, we upregulated UMOD expression in NRK-52E cells which was verified by qRT-PCR and Western blot. The remarkable elevation of UMOD expression determined in mRNA (Figure 2(d), *p* < 0.001) and protein levels (Figure 2(e), *p* < 0.001) signified the successful transfection. Furthermore, the results of cell-surface biotinylation assay revealed that TRPV5 was much in abundance on the surface of NRK-52E cells cotransfected with UMOD and TRPV5 overexpression, compared with its negative control (Figure 3(a)).

### 3.3. UMOD Regulated the Protecting Role of TRPV5 in Oxalate-Induced Cells

(Figure 3(a)). In order to unravel the interplay of UMOD and TRPV5 in oxalate-induced NRK-52E cells, we conducted siUMOD transfection. QRT-PCR determined that UMOD mRNA is expressed at a low level in siUMOD-transfected cells (Figure 3(b), *p* < 0.001), and the similar result in its protein level was measured by Western blot (Figure 3(c), *p* < 0.001). Subsequently, MTT and crystal adhesion assays demonstrated that siUMOD notably offset the promoting effect of TRPV5 on cell viability (Figure 3(d), *p* < 0.01) and the inhibiting effect of TRPV5 on the CaOx adhesion (Figure 3(e)) in NRK-52E cells induced by oxalate. Besides, we measured the Ca^2+^ content after divergent transfections. As results described in Figure 3(f), TRPV5 markedly restrained oxalate-induced reduction in intracellular Ca^2+^ concentration of NRK-52E cells, which was partially restored with the addition of siUMOD (*p* < 0.001).

### 3.4. miR-103a-3p Targeted UMOD and Is Mediated in the Regulation of the UMOD/TRPV5 Axis in Oxalate-Induced Cells

**Figure 4:**
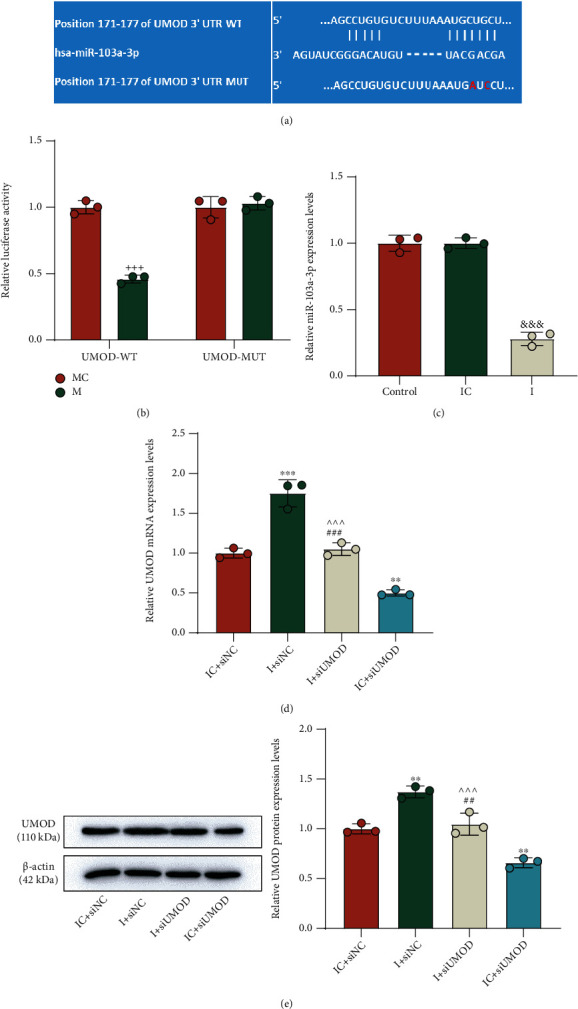
miR-103a-3p inversely regulated UMOD expression in NRK-52E cells. (a) The TargetScan website was used to predict binding sites between miR-103a-3p and UMOD. (b) Dual-luciferase report assay was carried out to validate that UMOD was targeted by miR-103a-3p. (c) QRT-PCR was performed to detect miR-103a-3p expression following the treatment with the miR-103a-3p inhibitor in NRK-52E cells. U6 was used as the internal control. (d) The effect of the miR-103a-3p inhibitor on UMOD expression in the cells with or without siUMOD was detected by qRT-PCR. *β*-Actin served as the internal control. (e) The effect of the miR-103a-3p inhibitor on the UMOD protein level in the cells with or without siUMOD was measured by Western blot. *β*-Actin served as the internal control. ^+++^*p* < 0.001 vs. MC; ^&&&^*p* < 0.001 vs. IC; ^∗∗^*p* < 0.01 and ^∗∗∗^*p* < 0.001 vs. IC + siNC; ^∧∧∧^*p* < 0.001 vs. IC + siUMOD; ^##^*p* < 0.01 and ^###^*p* < 0.001 vs. I + siNC. UMOD: uromodulin; qRT-PCR: quantitative real-time polymerase chain reaction; NC: negative control; siUMOD: small interfering RNA against UMOD; I: inhibitor; IC: inhibitor control.

**Figure 5:**
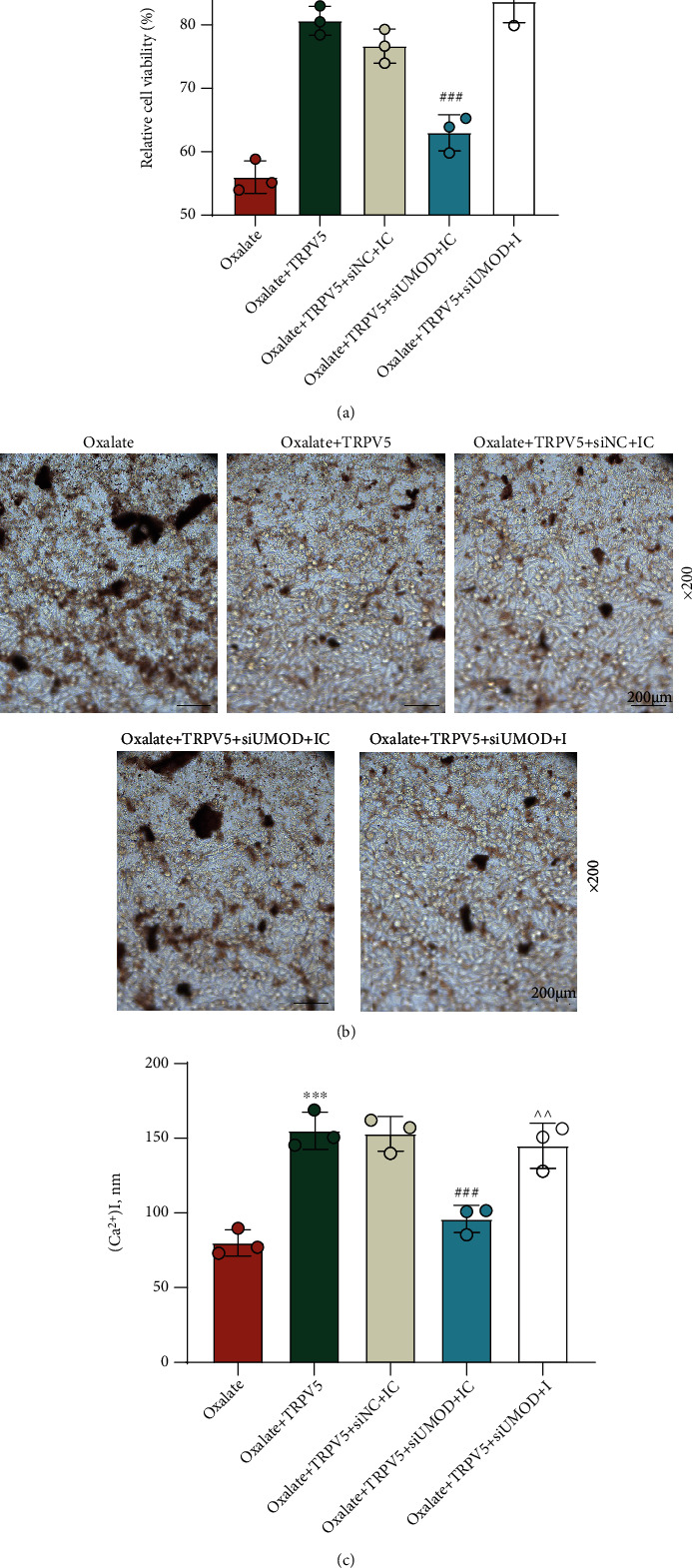
miR-103a-3p silencing activated the UMOD/TRPV5 axis to attenuate NRK-52E cells from oxalate-induced injury. (a) The effect of the miR-103a-3p inhibitor on cell viability in NRK-52E cells treated with oxalate, TRPV5-overexpressing plasmid, and siUMOD was determined by MTT assay. (b) Crystal cell adhesion assay was used to quantify CaOx deposition in specified cells (×200 magnification, scale bar = 200 *μ*m). (c) The Ca^2+^ content measurement in different groups was analyzed by the Fluo-4 AM Kit. ^∗∗∗^*p* < 0.001 vs. oxalate; ^###^*p* < 0.001 vs. oxalate + TRPV5 + siNC + IC; ^∧∧^*p* < 0.01 and ^∧∧∧^*p* < 0.001 vs. oxalate + TRPV5 + siUMOD + IC. TRPV5: transient receptor potential cation channel subfamily V member 5; UMOD: uromodulin; MTT: methylthiazolyldiphenyl-tetrazolium bromide; NC: negative control; siUMOD: small interfering RNA against UMOD; I: inhibitor; IC: inhibitor control.

Under the assistance of the TargetScan website, the result of gene prediction manifested that miR-103a-3p may contain binding sites for UMOD (Figure 4(a)). Then, luciferase assay detected the suppressed luciferase activity in the cells cotransfected with miR-103a-3p mimic and UMOD-WT (Figure 4(b), *p* < 0.001). To fathom out the regulating effect of miR-103a-3p on UMOD, we implemented miR-103a-3p inhibition in NRK-52E cells, with qRT-PCR detection confirming that the expression of miR-103a-3p was strongly downregulated after the transfection of the miR-103a-3p inhibitor (Figure 4(c), *p* < 0.001). Interestingly, the mRNA expression of UMOD was proved to be elevated after miR-103a-3p inhibition, which was visibly overturned with the addition of UMOD silencing (Figure 4(d), *p* < 0.01). By detection of Western blot, the alteration of UMOD protein expression was found to be the same as those occurring in UMOD mRNA expression (Figure 4(e), *p* < 0.01). Moreover, we conducted rescue experiments regarding cell functions. As demonstrated, the regulating role of the UMOD/TRPV5 axis in cell viability (Figure 5(a), *p* < 0.001), CaOx adhesion (Figure 5(b)), and intracellular Ca^2+^ concentration (Figure 5(c), *p* < 0.01) was strikingly reversed by the miR-103a-3p inhibitor.

### 3.5. miR-103a-3p Silencing Mitigated EG-Induced CaOx Deposition in Kidney Tissues *In Vivo* by Activating the UMOD/TRPV5 Axis

**Figure 6:**
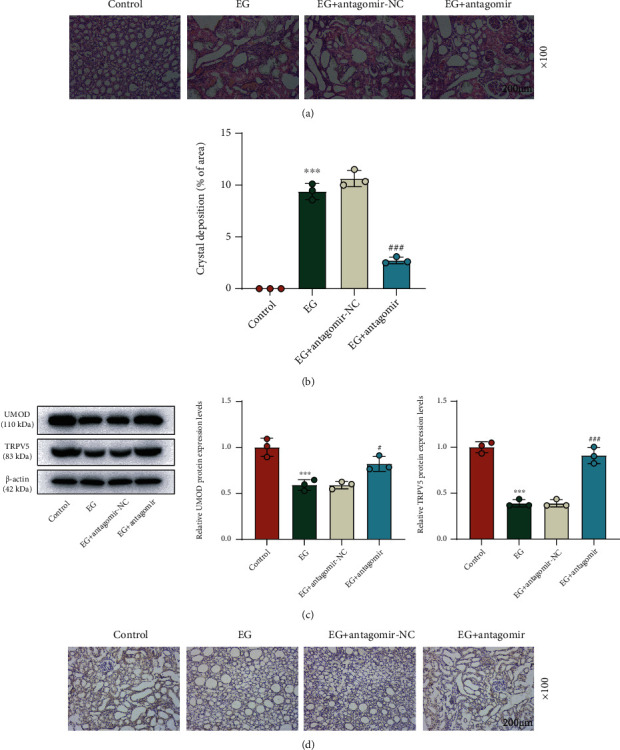
miR-103a-3p silencing mitigated EG-induced CaOx deposition in kidney tissues *in vivo* by activating the UMOD/TRPV5 axis. (a, b) CaOx deposition was measured by H&E staining in kidney tissues of rat treated with EG or EG and miR-103a-3p antagomir (×100 magnification, scale bar = 200 *μ*m). (c) Western blot was used to measure the protein levels of UMOD and TRPV5 in kidney tissues of rat treated with EG or EG and miR-103a-3p antagomir. *β*-Actin served as the internal control. (d) IHC analysis of TRPV5 expression was performed in the tissues of different groups (×100 magnification, scale bar = 200 *μ*m). ^∗∗∗^*p* < 0.001 vs. control; ^#^*p* < 0.05, ^###^*p* < 0.001 vs. EG + antagomir-NC. HE: hematoxylin and eosin EG, ethylene glycol; TRPV5: transient receptor potential cation channel subfamily V member 5; UMOD: uromodulin; NC: negative control; IHC: immunohistochemistry.

For further bolstering our findings *in vitro*, we constructed the hyperoxaluria rat model for the *in vivo* study. As illustrated in Figures 6(a) and 6(b), substantial deposition of CaOx crystals in kidney tissues of the EG group was observed comparing to that in the control group and this trend was reversed by miR-103a-3p antagomir (*p* < 0.001). Then, the results of Western blot indicated that EG strongly downregulated both UMOD and TRPV5 protein levels, which was markedly restored by miR-103a-3p antagomir (Figure 6(c), *p* < 0.05). By IHC, we noticed that TRPV5 expression was evidently downregulated in the EG group compared to the control group and it was upregulated in the EG + antagomir group compared to the EG + antagomir-NC group (Figure 6(d)).

## 4. Discussion

In the context of increasingly diverse surgical treatments against kidney stones, what should be underscored lies in prominent methods to prevent its occurrence and recurrence [27]. The most common component of kidney stones is CaOx crystals. There are many factors that influence the development of Ca^2+^-contained kidney stones, with hypercalciuria being by far the main recognized risk factor [28]. Tsuruoka et al. demonstrated that through the renal tubules, reduced Ca^2+^ reabsorption is instrumental in the induction of hypercalciuria and that active calcium reabsorption could be regulated by various factors [29]. Later, Worcester et al. proposed that postprandial urinary Ca^2+^ levels were starkly higher in patients with hypercalciuria compared to the controls, even when blood parathyroid hormone secretion and renal load were at the same level [30]. Collectively, it is connoted that impaired renal tubular reabsorption may be the foremost reason for hypercalciuria. This study therefore explored the underlying molecular mechanism that regulates urinary Ca^2+^ levels from the perspective of Ca^2+^ reabsorption, so as to put forward momentous channels to prevent and treat kidney stones.

TRPV5 acts as a renal tubular epithelial Ca^2+^ channel that mediates Ca^2+^ transport and reabsorption in the kidney [31]. Jang et al. pointed out that TRPV5 protein expression was overtly downregulated in the renal tissues of hypercalciuric rats but was restored by hydrochlorothiazide administration, indicating that the mechanism of hydrochlorothiazide treatment for hypercalciuria may be achieved through upregulation of TRPV5 protein expression in the renal tissues [32]. Studies have validated that high oxaluria induces reactive oxygen species (ROS) production, damages renal tubular epithelial cells, and alters the structure of phosphatidylserine in the cell membrane, leading to adhesion of CaOx to the surface of renal tubular epithelial cells [33]. In the previous research, we uncovered that remarkably downregulated TRPV5 appeared after inducing NRK-52E cell injury by oxalate, the trend of which was reversed after transfection of TRPV5 overexpression. Additionally, upregulation of TRPV5 enhanced cell viability and inhibited CaOx adhesion in NRK-52E cells induced by oxalate, indicating that TRPV5 generates a critical preventive effect on oxalate-induced CaOx stone formation in the kidney.

As a primary channel for Ca^2+^ reabsorption, the activity and expression of TRPV5 could be regulated by multiple factors [34]. For instance, a recent study confirmed that UMOD prevents the formation of kidney stones by stimulating Ca^2+^ reabsorption via TRPV5 [35]. Moreover, it was noted that 87.5% of mice with UMOD knockout formed calcium oxalate and calcium phosphate stones in the CNT, renal medulla, and papillae at 15 months of age. All these findings suggested that UMOD could interact with TRPV5 in the formation of kidney stones, which has not been borne out yet. However, our study identified for the first time the existence the interaction that UMOD positively regulated TRPV5 expression functioning in injured NRK-52E cells by strengthening cell viability, reducing CaOx adhesion, and enhancing the reabsorption of Ca^2+^.

miRNAs are involved in the development and progression of kidney diseases through their targeted regulation of mRNAs. miR-21 was found to contribute oxalate-induced renal tubular cell injury by upregulating PPARA [36]. In addition, Jiang et al. reported that inhibiting miR-155-5p to upregulate MGP expression could observably attenuate oxalate-induced oxidative stress injury in the kidney [37]. However, few studies have devoted to ascertaining the mechanism of miRNA regulation on UMOD affecting kidney stone formation. In order to gain a further insight into the pathogenesis of this disease, this study employed bioinformatics analysis and luciferase assay to find the miRNA, miR-103a-3p, potentially regulating UMOD. In renal disease research, the miR-103a-3p cycle has been confirmed to make profound impacts upon renal inflammation and fibrosis [38]. Peters et al. also suggested that miR-103a-3p could be regarded as a potential biomarker in chronic kidney disease [39]. In our study, interestingly, we verified the molecular mechanism of miR-103a-3p in kidney stones that miR-103a-3p silencing could alleviate oxalate-induced injury in NRK-52E cells by activating the UMOD/TRPV5 axis. Furthermore, we proved this regulatory mechanism even more strongly through *in vivo* experiments in EG-induced rat models. However, there is a lack of consideration in this study as it involves a single type of cell and a relatively small number of study variables in the *in vitro* experiments.

## 5. Conclusions

Overall, our current study unraveled that miR-103a-3p silencing ameliorates CaOx deposition in the rat kidney by activating the UMOD/TRPV5 axis, which lays a novel theoretical foundation for prevention, diagnosis, and treatment strategies towards kidney stones from the perspective of Ca^2+^ reabsorption.

## Figures and Tables

**Table 1:** The primer sequences used for qRT-PCR.

	Forward (5′-3′)	Reverse (5′-3′)
TRPV5	TGGTGGGTCAGAGACCAAGA	GCAGCAAAGGACAAAGGGTG
UMOD	CCAACACACAGGTGGCATTG	CTCCGATGGGTGGGTTTTGA
*β*-Actin	GGCTGTATTCCCCTCCATCG	CCAGTTGGTAACAATGCCATGT
miR-103a-3p	CGCGAGCAGCATTGTACAGGG	AGTGCAGGGTCCGAGGTATT
U6	CTCGCTTCGGCAGCACA	AACGCTTCACGAATTTGCGT

## Data Availability

The analyzed datasets generated during the study are available from the corresponding author upon reasonable request.
